# Physiological and Molecular Mechanisms of Plant Responses to Copper Stress

**DOI:** 10.3390/ijms232112950

**Published:** 2022-10-26

**Authors:** Guang Chen, Jia Li, Huimin Han, Ruiying Du, Xu Wang

**Affiliations:** 1 Institute of Quality Standard and Monitoring Technology for Agro-Products of Guangdong Academy of Agricultural Sciences, Guangzhou 510640, China; 2 Key Laboratory of Testing and Evaluation for Agro-Product Safety and Quality, Ministry of Agriculture and Rural Affairs, Guangzhou 510640, China; 3 Guangdong Provincial Key Laboratory of Quality & Safety Risk Assessment for Agro-Products, Guangzhou 510640, China; 4 State Key Laboratory of Cotton Biology, Institute of Cotton Research of Chinese Academy of Agricultural Sciences, Anyang 455000, China

**Keywords:** copper toxicity, absorption and transport, copper homeostasis, tolerance mechanism

## Abstract

Copper (Cu) is an essential micronutrient for humans, animals, and plants, and it participates in various morphological, physiological, and biochemical processes. Cu is a cofactor for a variety of enzymes, and it plays an important role in photosynthesis, respiration, the antioxidant system, and signal transduction. Many studies have demonstrated the adverse effects of excess Cu on crop germination, growth, photosynthesis, and antioxidant activity. This review summarizes the biological functions of Cu, the toxicity of excess Cu to plant growth and development, the roles of Cu transport proteins and chaperone proteins, and the transport process of Cu in plants, as well as the mechanisms of detoxification and tolerance of Cu in plants. Future research directions are proposed, which provide guidelines for related research.

## 1. Introduction

Heavy metal poisoning is currently a worldwide environmental problem. It is primarily caused by human activities, such as mining, the discharge of waste gas, irrigation with wastewater, and the use of products that contain heavy metals that exceed the allowable concentration. Heavy metals can cause serious effects on the environment and living organisms, even at trace concentrations. Among the heavy metals, copper is one of the eight micronutrients that is essential for plant growth and related to many physiological and biochemical processes in plants. As a cofactor for many enzymes, such as laccase, cytochrome c oxidase, polyphenol oxidase, copper/zinc superoxide dismutase (Cu/Zn-SOD), amino oxidase, and phycocyanin [1], copper (Cu) plays a key role under stress conditions. Cu is also associated with oxidative phosphorylation, protein trafficking, signal regulation, and lipid and iron metabolism [2]. Therefore, Cu is a nutrient that is essential to the regular metabolism of plants. Plants develop various abnormal phenotypes when they are grown under Cu-deficient conditions, including stunted growth and reproductive development, twisted young leaves, and insufficient water transport [3]. However, excessive Cu can also adversely affect plant growth and metabolism; seriously interfere with plant growth and development and nutrient absorption; inhibit photosynthesis, root development, and leaf extension; and affect the functions of some key cellular components, such as proteins, lipids, DNA, and RNA [4]. Elucidating the biochemical activities of Cu in soil-plant systems is critical to maintaining regular plant development and sturdy growth.

## 2. The Biological Functions of Copper

Cu is one of the essential trace elements that are necessary to maintain the regular growth and development of plants. As a cofactor, it is the active center of various enzymes and is widely involved in numerous biological activities, such as protein transport, cell wall metabolism, respiration/photosynthesis electron transfer, and hormone signal transduction [4]. Cu deficiency leads to the attenuation of plant growth, distortion or the yellowing of young leaves (chlorosis), curling of the leaf margins, damage to the apical meristems, and decreased seed setting rates [5,6]. Cu deficiency in forests seriously affects wood production [7]. The secondary effects of Cu deficiency may be insufficient water transport due to reduced cell wall formation and lignification in some tissues, such as the xylem [5]. Cu deficiency has a severe impact on pollen and embryonic development, pollen and seed viability, and seed and fruit production [8]. Cu-associated proteins are essential in the electron transport chain of chloroplast and mitochondria. Cu participates in the photosynthetic reaction of PSII independent of plastocyanin and stimulates the oxygen-evolving activity of PSII in vitro [9,10].

Cu is required for Cu/Zn-SOD [11], ethylene receptor [12], laccase, polyphenol oxidase, and other multicopper oxidases [13]. Some members of copper oxidases, such as amine oxidase enzymes, which bind to cell walls, catalyze the oxidation of putrescine to produce hydrogen peroxide (H_2_O_2_) required for lignification, cross-linking of cell wall protein, and programmed cell death [14]. H_2_O_2_ is a signaling molecule that is involved in various physiological and biochemical processes, and it can regulate plant growth and development, activate plant stress resistance and stress tolerance, strengthen cell walls, improve photosynthesis, delay senescence, and enhance stomatal movement [1].

## 3. Cu Toxicity

### 3.1. Soil Contamination

Cu exists in various forms in the soil, such as Cu oxide, carbonate, sulfate, and sulfide. Under natural conditions, the average concentration of Cu in the soil is 6–80 mg kg^−1^. Owing to human activities, particularly industrial and agricultural production, the concentrations of Cu in the soil have increased dramatically, and it is now considered to be an important pollutant. The extensive use of agrochemical products that contain Cu, such as fertilizers, fungicides, herbicides, and pesticides, can also lead to the accumulation of Cu in soil [15]. Cu that has accumulated in the soil cannot be degraded biologically or chemically and poses a threat to the environment, food security, and human health. High concentrations of Cu (20–100 mg kg^−1^) can have toxic effects on soil microorganisms and hinder the mineralization of nutrients, such as phosphorus (P) and nitrogen (N); the presence of excessive amounts of Cu reduces the availability of P [16]. Moreover, the levels of other trace elements, such as iron and zinc, can decrease as the Cu accumulates. Owing to the low mobility and solubility of Cu, the resultant soil pollution can persist for many years [17].

### 3.2. Root Damage

Cu toxicity first occurs in the roots and then expands to the aboveground parts, affecting various physiological processes. High concentrations of Cu in the soil can limit and impair root growth, which results in the reduced uptake of nutrients and water. Typically, the inhibition of root growth is related to the rupture of the root epidermis and exodermis. Cu toxicity leads to the rupture of root cuticles, a reduction in the proliferation of root hairs, darker color, stunted growth, and severe deformation of the root structure [18]. Studies have shown that Cu ions can change the rate of proliferation of root meristem cells by regulating hormones, such as melatonin, auxin, and abscisic acid, in plant root cells, thereby affecting root development [19,20]. Batool et al. [21] reported that the inhibition of root growth is associated with a decrease in cell division, which results in an increase in cell wall thickness. Marques et al. [18] found that high concentrations of Cu (1000 mg L^−1^) changed the root morphology, root volume, and the number of root hairs of Siberian cypress (*Microbiota decussata*).

### 3.3. Nutrient Deficiency

Large amounts of Cu in the soil can inhibit the uptake of potassium and P [22], primarily because of ion antagonism and the inhibition of root growth. Hippler et al. [23] found that high concentrations of Cu reduced the uptake of N and its accumulation in plants, and the reduction in Cu-mediated N uptake is primarily owing to the downregulated levels of expression of the nitrate reductase NR1, low-affinity nitrate transporters (NRT1 family), and the *bZIP* transcription factors, such as *TGA1* and *TGA4*, that regulate nitrate transporters. In addition, high concentrations of Cu in citrus reduced the levels of N, manganese, zinc, and calcium in the roots and calcium, iron, and manganese in the leaves [24]. The contents of Cu, P, and sulfur in alfalfa (*Medicago sativa*) increased significantly when the concentration of Cu exceeded 5.0 mg L^−1^, while the contents of P and iron in lettuce (*Lactuca sativa*) decreased correspondingly [25]. The uptake of iron, zinc, calcium, and manganese by sorghum (*Sorghum bicolor*) roots decreased significantly with the increase of concentration of Cu [26].

### 3.4. Inhibition of Photosynthesis

Excessive Cu interferes with the composition of chloroplast and thylakoid membranes, which can induce oxidative stress in plant cells and reduce the contents of photosynthetic pigments and electron carriers, thereby inhibiting electron transfer in photosynthesis [27,28]. Leaf yellowing is one of the primary signs of Cu poisoning, and Hossain et al. [29] observed a significant decrease in the content of photosynthetic pigments, such as chlorophyll a, chlorophyll b, and carotenoids, after lentils (*Lens culinaris*) were treated with 3 mM of Cu sulfate (CuSO_4_). Panou-Filotheou et al. [30] reported that Cu toxicity (17–25.5 mM) significantly reduced the volume and number of chloroplasts. In addition, the grana thylakoids degenerate and swell under Cu stress, and there is often a high content of globulin in the plastids.

Cu plays an important role in plant photosystem II (PSII)-mediated electron transport, which is involved in the photolysis of water molecules in photosynthetic cells. However, high concentrations of Cu can affect the efficacy of light-harvesting complex II (LHCII) or PSII. High concentrations of Cu in sea buckthorn (*Hippophae rhamnoides*) primarily affected photosynthesis by inhibiting the PSII reaction center. Treatment with 23 mM of Cu inhibited the activity of PSII by directly reducing the content of chlorophyll, which resulted in inefficient photosynthesis [31]. Cu inhibited the electron transport of PSII at a concentration of 75–150 μM, which, in turn, affected the composition of the thylakoid membrane of black algae (*Audouinella* spp.) [32]. Therefore, excessive amounts of Cu can reduce the photosynthesis of plants by inhibiting chlorophyll biosynthesis and PSII, thus, adversely affecting the plants. Therefore, it is crucial to strictly control the equilibrium of Cu in plants.

## 4. Absorption and Transport of Copper in Plants

Plants exhibit different physiological responses to Cu toxicity. Thus, maintaining the homeostasis of Cu ions in plants is a delicate regulatory process. Various Cu transporters play an important role in the entire regulatory process by participating in key activities, such as the absorption, chelation, transport, and compartmentalization of Cu. Cu transporters can be divided into two categories based on their different functions. One is composed of uptake transporters, which are responsible for the transportation of extracellular Cu ions into cells. The other is composed of efflux transporters, which transport intracellular Cu ions to extracellular spaces or into organelles. The transport process of Cu ions is primarily divided into the following four steps: (1) absorption by the roots; (2) isolation in the vacuoles; (3) loading in the xylem and phloem; and (4) distribution and redistribution of the nodes [33,34]. Different transporters are required for these processes. The Cu transporters that have been cloned include heavy metal ATPase (HMA), zinc-regulated transporters (ZRTs), iron-regulated transporters (IRTs), and yellow stripe-like transporters (YSLs) [35]. Studies have shown that these transporters, pumps, and channels help plants to absorb and transport minerals (Table 1) and maintain intracellular Cu ions in a relatively homeostatic state, which can not only ensure regular plant growth and development but also respond to environmental changes [36].

### 4.1. Copper Transporters

#### 4.1.1. Heavy Metal ATPases

P_1B_-ATPases are a family of transporters that transport heavy metal ions across the membranes by hydrolyzing ATP [60], and they are also known as heavy metal transporting ATPase (HMA). The structure of P_1B_-ATPases typically includes eight transmembrane domains (M1–M8), three functional domains (A, P, and N), and N- and C-terminal soluble metal binding domains (MBDs) [61]. The A actuator domain is an ATP binding domain, which is located between M2 and M3; the P functional domain is a phosphorylation domain, and the N functional domain is a nucleotide-binding domain, which is located between M3 and M4 [62].

The dicotyledonous model plant Arabidopsis was utilized as an example. It contains eight HMA members, i.e., AtHMA1–AtHMA8. AtHMA1, AtHMA6, and AtHMA8 are high-affinity Cu transporters located on the chloroplast. They are responsible for the transport of Cu in the chloroplast. In addition, they transport Cu to the chloroplast stroma and thylakoid to synthesize plastocyanin and also provide cofactors for Cu/Zn-SOD in the stroma [42,63]. AtHMA6 transports cytosolic Cu to the stroma, while AtHMA8 transports Cu to the thylakoid lumen [63]. Hussain et al. [64] found that the photosynthesis of Arabidopsis mutants *athma6* and *athma8* was affected, indicating that AtHMA6 and AtHMA8 maintain photosynthetic stability in plants. The accumulation of Cu in the *athma1* loss-of-function mutant was significantly lower than that in the wild type, and the Cu/Zn-SOD activity in chloroplasts was inhibited. However, the content of plastocyanin was normal. These results indicate that AtHMA1 can transfer Cu^2+^ and Zn^2+^ to the plasto-Cu/Zn-SOD [65,66].

AtHMA7 is localized to the endoplasmic reticulum and primarily expressed in roots and flowers. AtHMA7 regulates ethylene signal transduction by interacting with trans-cyclooctene, an ethylene antagonist [43]. Li et al. [67] found that ethylene receptors are Cu-dependent proteins, and the expression of *AtHMA7* in Arabidopsis was significantly altered after Cu treatment [68], demonstrating that *AtHMA7* is induced by Cu. Woeste et al. [69] revealed that *AtHMA7* acts upstream of the ethylene receptor gene family through an analysis of genetic pathways. *AtHMA5* is regulated by higher concentrations of Cu and transports surplus Cu to the xylem via the symplast. Increased contents of Cu were observed in the roots of an *AtHMA5* knockout mutant, which was sensitive to Cu [41]. In cucumber (*Cucumis sativus*), the level of expression of *CsHMA5.2* increases under Cu stress, which promotes the accumulation of Cu [70]. Unlike CsHMA5.2, the plasma membrane-localized rice (*Oryza sativa*) OsHMA5 is associated with the efflux of metals.

#### 4.1.2. Copper Transporter Proteins (COPTs)

The COPT transporter has a sequence that is similar to that of the copper transporter of eukaryotic cells and belongs to the Cu transporter (CTR) family, which consists of six members, COPT1–COPT6, that have been identified in Arabidopsis. This family consists of three transmembrane domains (TMDs). TMDs have a His- and/or Met-rich domain, and the conserved motifs MXXXM and GXXXXG are linked to TMD2 and TMD3. They can be divided into three groups based on the number of their domains. The first includes three Met- and His-rich domains, i.e., COPT1, COPT2, and COPT6 [71]_._ The difference between the three is that COPT1 and COPT2 have a CXC motif at the C-terminus, while COPT6 lacks that. The proteins of this group have a high affinity for Cu and a strong transport capacity, which is hypothesized to be responsible for the ability of Cu ions to flow in and out of cells. The second group includes COPT3 and COPT5, which have only one Met- and His-rich domain. They lack a high affinity for Cu and cannot transport Cu as efficiently, which could be responsible for the transport of Cu ions into and out of cells. The third group is COPT4, which lacks the Met residues and MXXXM motif required to mediate the transport of Cu ions, and it could function by interacting with the other COPT proteins [72].

The COPT1 transporter is responsible for the uptake of Cu and is primarily distributed on the plasma membrane of the root tip. When Cu is deficient, plants upregulate the expression of *COPT1*, thereby acquiring large amounts of Cu from the growth substrate [48]. The absorption of Cu by plants generates hydroxyl radicals (OH^−^). After OH^−^ combines with the non-selective cationic channels on the plasma membrane, the calcium ion channel is opened, thereby promoting root growth [73]. The level of expression of *COPT2* is also induced by Cu deficiency [49], and it is expressed at even higher levels when Cu and iron are deficient. When the plants are deficient in phosphorous (P), COPT2 is involved in P signal transduction by delivering Cu to Cu proteins that respond to low P signals [49]. COPT6 is also localized to the plasma membrane and is primarily expressed in the vascular tissues and reproductive organs of Arabidopsis. Its amino acid sequence is highly similar to those of COPT1 and COPT2 [71] and is involved in the redistribution of Cu from vegetative to reproductive organs [74]. Low treatment with Cu significantly induces the level of expression of *COPT6* in Arabidopsis. Compared with wild-type Arabidopsis under Cu deficient conditions, the content of Cu is increased in the *COPT6* mutant rosette leaves, while the content of Cu in seeds is decreased.

There are fewer studies on COPT3 and COPT5. Bock et al. [75] found that *COPT3* is expressed in early-developing pollen, the vascular bundles of leaves, and stamen filaments. In Arabidopsis leaves in which COPT3-HA is expressed, the isolation of membrane fractions using the sucrose density gradient technique identified that the distribution of COPT3 is similar to that of the endoplasmic reticulum marker protein SEC12. Therefore, it is hypothesized that COPT3 is localized to the endoplasmic reticulum [76]. COPT5 is a tonoplast-localized protein, which is highly expressed in the endothelium, vascular bundles, and root hairs of the main root but is relatively weakly expressed in the shoots; it is mostly located in the vascular bundles of the hypocotyl, cotyledons, and leaves [77,78]. The phenotype of the *COPT5* mutant under normal conditions does not differ from that of the wild-type, but under Cu deficiency treatment, growth of the shoots and roots is inhibited, which reduces the content of chlorophyll [78]. In addition, *COPT5* mutants are also sensitive to cadmium stress, suggesting that Cu transport plays an important role in the resistance of plants to cadmium stress [79]. COPT4 is not directly involved in the transport of Cu ions, and its specific function merits further study.

#### 4.1.3. ZIP

The ZIP (Zrt, Irt-like protein) family is composed of two types of members—zinc-regulated transporter (ZRTs) and iron-regulated transporters (IRTs) [47]. Members of the ZIP family primarily maintain the intracellular ion balance by transporting various metal cations, such as zinc, iron, Cu, and cadmium, into the cytoplasm [48]. Most ZIP proteins have eight transmembrane domains, and the N- and C-termini are located on the outer surface of the plasma membrane and contain 309–476 amino acids. This variation in length is primarily determined by the distance between the transmembrane domains III and IV, known as the “variable region.” This region is rich in histidine residues, which are possibly related to the binding and transport of metal ions [47]. ZIP proteins are widely found in fungi, bacteria, animals, and plants, and more than 100 types have been identified, including 11 in Arabidopsis (AtZIP1–AtZIP11) and 17 in rice (OsZIP1–OsZIP17).

Cu deficiency in Arabidopsis induces the upregulated expression of *AtZIP2* and *AtZIP4*, whereas Cu stress represses their expression [80]. AtZIP2 and AtZIP4 can restore the phenotype of the Cu uptake-deficient yeast mutant *MPY17*. In rice, Cu stress induces the expression of *OsZIP1*, and complementation assays in yeast (*Saccharomyces cerevisiae*) showed that OsZIP1 reduces the accumulation of metals. Under Cu stress, transgenic rice lines that overexpress *OsZIP1* accumulate less Cu in vivo compared with the wild type. In contrast, *oszip1* mutants and RNA interference (RNAi) lines accumulate more Cu in the roots. *OsZIP1* could act as a Cu transporter to function in the response of rice plants to Cu stress [81]. However, the functions of most ZIP proteins merit further investigation.

#### 4.1.4. YSL Proteins 

The YSL protein family is primarily involved in the transport of heavy metals in Gramineae plants and is responsible for the long-distance transport of nicotianamine and phytosiderophores [35]. In Gramineae plants, a large quantity of mugine acids (MAs) is synthesized, and they are secreted to the roots to chelate with Fe^3+^ and form complexes. YSL proteins transport them into the cells to maintain iron homeostasis in vivo.

Arabidopsis has been found to have eight YSL family members (AtYSL1–AtYSL8), and rice has 18 members (OsYSL1–OsYSL18). In Arabidopsis, AtYSL2 is involved in the transport of Fe (II)-NA and Cu-NA, and its transcriptional level is regulated by iron and Cu [46]. The E3 ubiquitin ligase SIZ1 can regulate the expression of *AtYSL1* and *AtYSL3*, which are involved in maintaining Cu homeostasis and improving the tolerance of plants to Cu [82]. Rice OsYSL16 is primarily responsible for transporting the Cu-NA complexes through the phloem to nascent tissues and seeds [51,83]. In *OsYSL16* knockout lines, there was a significant reduction in the transport of Cu-NA from the old leaves to new leaves and from the flag leaves to panicles, and the administration of Cu can improve the rate of pollen germination in the *OsYSL16* mutant [83].

#### 4.1.5. Natural Resistance-Associated Macrophage Proteins (NRAMPs)

NRAMP proteins are widely present in organisms, such as bacteria, yeast, plants, mice, and humans, and this gene family plays a key role in the transport of divalent metal ions across cell membranes [84]. Li et al. [85] found that treatment with 50 μM CuCl_2_ significantly increased the levels of expression of *HvNramp1*, *HvNramp2*, *HvNramp5*, and *HvNramp9* in barley (*Hordeum vulgare*), suggesting that NRAMP could be involved in the transport of Cu ions in barley. Chou et al. [86] found that Cu stress induces the expression of *M189530_c0*, which is homologous to *OsNramp5*, which could be involved in the transport of Cu. However, the mechanism by which NRAMP proteins regulate Cu transport in plants merits further study.

### 4.2. Copper Chaperones

The movement of copper between cells in plants is achieved by Cu chaperones [87]. Cu chaperones are a class of metal receptor proteins with low molecular weight, which are widely present in the cells of various organisms and contain a Cu ion-binding domain. This domain is responsible for binding the Cu ions and delivering them to specific Cu transporters. Cu chaperones prevent Cu from interacting with other intracellular components [88], thus avoiding the toxicity of free Cu ions. Various types of Cu chaperones, such as CCH, CCS, and COX17, have been identified in plants. Among them, the *CCH* gene in Arabidopsis has been studied the most thoroughly [89].

To date, the reported Cu chaperones in Arabidopsis include Cytochrome C Oxidase17 (AtCOX17), Cu Chaperone for SOD (AtCCS), Antioxidant Protein1 (AtATX1), and ATX1-like Cu Chaperone (AtCCH) [90]. AtCCH and AtATX1 have high sequence homology, and both contain Lys residues, βαββαβ folded structures, and N-terminal MXCXXC Cu ion binding domains, which are involved in intracellular Cu transport and the scavenging of reactive oxygen species (ROS) [91]. In addition, AtCCH can shuttle between the sieve tubes and companion cells, possibly owing to its unique C-terminal extension sequence [89]. Cu deficiency induces the expression of *AtCCH*, while excessive amounts of Cu inhibit its expression. The expression of *AtATX1* increases under Cu stress. The overexpression of *AtATX1* significantly increases the accumulation and tolerance of Cu in the shoots of Arabidopsis [92,93]. AtCOX17 is homologous to the yeast COX17 protein and can transport Cu to cytochrome C oxidase that is located in the mitochondria. The heterologous expression of *AtCOX17* complements the phenotype of the yeast respiration-deficient mutant strain *Δcox17* [94]. AtCCS is a chaperone protein that is localized to both the cytoplasm and plastids [90], with a chloroplast targeting sequence; therefore, the function of AtCCS is to deliver Cu ions to Cu/Zn-SOD located in the cytoplasm or plastids [95].

## 5. Mechanisms of Copper Detoxification and Tolerance in Plants

The mechanism of Cu detoxification and tolerance in plants primarily occurs through the induction of the expression of specific functional genes and the production of antioxidants [96]. Under Cu stress, a large amount of ROS will be produced in plants, and there are three mechanisms to maintain the optimal level of Cu and ROS homeostasis [97]. The first is to reduce or prevent the roots from absorbing Cu by chelating or precipitating Cu ions by plant root exudates. The second is to induce the expression of Cu absorption and transport-related genes to reduce the concentration of intracellular Cu ions, thereby controlling Cu-mediated ROS production. Finally, different types of antioxidants remove surplus ROS to diminish the toxic effects of Cu.

### 5.1. Root Exudates

Root exudates refer to various substances secreted by plants into the growth substrates through the root system during the growth process. The root exudates can change the physical, chemical, and biological properties of the rhizosphere and improve or alleviate the growth state of plants under heavy metal poisoning conditions, which enables plants to adapt to changes in the external environment. Root exudates are roughly divided into three categories: (1) substances that diffuse or leak from root cells, such as sugars, amino acids, and vitamins; (2) metabolites that are actively secreted by root cells, such as enzymes, hormones, phenolics, and organic acids; and (3) substances that are produced by bacterial decomposition after the plant residues have been shed [98].

Lyubenova et al. [99] found an increase in the contents of oxalic acid, malic acid, acetic acid, and tartaric acid secreted by the roots of broadleaf cattail (*Typha radix*) under Cu stress. The content of Cu ions in the plant tissues was proportional to the content of organic acids with low molecular weights in the rhizosphere soil. Heavy metals can also form stable compounds with functional groups, such as amino, carboxyl, and hydroxyl, of histidine and proline (Pro), which aggressively detoxifies them [100]. An analysis of amino acid profiles showed that under Cu stress, the contents of free amino acids, such as Pro, cysteine (Cy), alanine, and aspartic acid, increased in mustard (*Brassica* spp.) seedlings [101]. A study by Zhu found that the exogenous application of β-aminobutyric acid activated the antioxidant enzyme system of tobacco (*Nicotiana* spp.), increased the content of glutathione, regulated the expression of metal ion transporter genes, and enhanced the ability of tobacco to resist Cu stress [102].

### 5.2. Ion Transport

**Figure 1:**
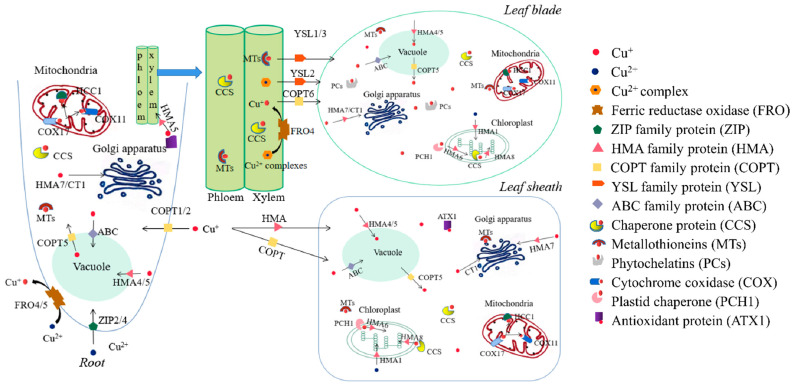
Copper transport process in plants. FRO, Ferric reductase oxidase; ZIP, Zn-regulated transporter; HMA, Heavy metal ATPase; COPT, Copper transporter protein; YSL, Yellow stripe-like protein; ABC, ATP-binding cassette transporter; CCS, Cu chaperone; MTs, Metallothioneins; PCs, Phytochelatins; COX, Cytochrome coxidase; PCH, Plastid chaperone; ATX1, Antioxidant protein; CT, MFS-type Cu transporter.

Plants improve their tolerance to Cu by transporting Cu ions into vacuoles or other organelles. Cu in the soil primarily exists in the form of compounds, namely Cu minerals, which form Cu^+^ and Cu^2+^ after weathering. Rhizosphere Cu^2+^ ions are reduced to Cu^+^ by the catalysis of ferric reductase oxidase 4/5. Cu^2+^ and Cu^+^ are transported into root cells by COPT1/2 and ZIP2/4, respectively, and then relocated to the mesophyll cells. Moreover, Cu^+^ from the external environment can be directly transported to leaf sheath cells by HMA and COPT [103]. In the cytoplasm, Cu^+^ is chelated by MTs or the two specific chaperones, CCS and ATX1, and transported to different organelles or sequestered in vacuoles to reduce the content of Cu in the cytoplasm. HMA4/5 and COPT5 control the transport of Cu^+^ from and into the vacuole, respectively, while HMA7 and CT1 (an MFS-type transporter) mediate the transport of Cu^+^ into the Golgi apparatus [103]. In mitochondria, COX17 in the intermembrane space delivers Cu^+^ to two chaperones, HCC1 and COX11, which are located on the inner mitochondrial membrane [104]. In fibrovascular tissues, Cu ions are transported in the forms of a Cu(I)-complex or Cu(II)-complex. ATX1 transmits Cu^+^ to HMA5, which loads Cu^+^ into the xylem. Complexed Cu^2+^ is transported into the leaves through YSL1/2/3 or reduced to Cu^+^ by ferric reductase oxidase 4 (FRO4), and Cu^+^ is then transported to the leaf cells by COPT6 [104]. Plastid Chaperone 1 (PCH1) and CCS are responsible for the allocation of Cu^+^ in the chloroplasts. PCH1 transfers Cu^+^ from the cytoplasm to the chloroplast membrane and then transports Cu^+^ to the Cu transporter HMA6 located in the inner membrane. HMA6 mediates the transport of Cu to the chloroplast stroma where Cu^+^ is bound by CCS and transferred to CSD2 and the Cu transporter HMA8, which is localized on the thylakoid membrane. Ultimately, HMA8 supplies Cu^+^ to plastocyanin in the thylakoid lumen [104,105] (Figure 1).

### 5.3. Antioxidative Enzymes

Under copper stress, antioxidant enzymes are crucial for scavenging ROS [106]. The activation and inactivation of antioxidant enzymes to respond to Cu-induced oxidative stress depend on ROS and the plant species [107]. It was found that three days of treatment with 50 μM CuCl_2_ significantly increased the contents of MDA and Pro and the activity of peroxidase (POD) in barley [85]. The activity of SOD in rice seedlings increased after treatment with 200 μM of Cu, and the activity of catalase (CAT) decreased [108]. After two concentrations of nano-Cu treatments, the ascorbic acid peroxidase (APX) and nitrate reductase (NR) activities in cowpea (*Vigna unguiculata*) leaves and roots and the activities of CAT in the roots were enhanced to varying degrees, while that activity of SOD decreased significantly, indicating that the response of cowpea to nano-Cu toxicity is specific to the organs [109]. Saleem et al. [110] found that with the increase of Cu concentration in soil, the activities of both SOD and POD in flax (*Linum usitatissimum*) showed an upward trend, and the enzyme activities reached their highest levels after treatment with 600 mg/kg Cu. Younis et al. [111] found that the activities of SOD and POD in common bean (*Phaseolus vulgaris*) were enhanced under low concentrations of Cu but decreased under high concentrations of Cu. In addition, in the same study, the activities of GR and APX were significantly enhanced under any concentration of Cu.

### 5.4. Non-Enzymatic Antioxidants

#### 5.4.1. Glutathione

Glutathione (GSH) is a low molecular weight tripeptide compound that contains a sulfhydryl group and is an important metal chelator and antioxidant [112], which facilitates the regulation of the cell cycle, antioxidant defenses, and cell detoxification. Through the ascorbic acid glutathione cycle (ASA–GSH), GSH improves the tolerance to Cu. Conte et al. [113] found that with the increase in concentration of Cu, the content of protein thiol in the cells of green algae *Scenedesmus* increased, while the content of GSH contrasted by significantly decreasing. In the same study, Cu stress was found to enhance the activities of glutamylcysteine synthase, glutathione S-transferase, and glutathione peroxidase and decrease the activity of glutathione reductase, which resulted in changes in GSH homeostasis. In addition, Mostofa et al. [114] found that GSH inhibited the absorption of Cu in rice, improved antioxidant activity, and alleviated Cu toxicity. In *Brassica napus*, GSH interacted with Cu and formed a complex with the Esh groups to enhance the resistance to Cu stress [115]. Thounaojam et al. [116] found that the content of GSH increased under Cu stress, and the tolerance of rice to Cu improved.

#### 5.4.2. Phytochelatins (PCs)

PCs are the most important metal chelators and play an important role in the homeostasis regulation of Cu ions. PCs are metal-chelating peptides catalyzed by PC synthase with GSH as substrates. PCs can chelate free metal ions to form compounds that are non-toxic or have very low levels of toxicity, thereby preventing the metal ions from being toxic to cells [117]. The Arabidopsis mutant *cad1-3* cannot synthesize PCs and displays enhanced sensitivity to metals [118]. Navarrete et al. [119] found that high concentrations of Cu induce the synthesis of PCs and produce stable Cu–PC complexes.

#### 5.4.3. Metallothioneins (MTs)

Metallothioneins are rich in Cys, and these proteins contain thiol groups and have a high ability to bind various metal ions, including Cu [107]. Therefore, they play an important role in the dynamic equilibrium of Cu in the cytoplasm. Liu et al. [120] found that the overexpression of rice *OsMT2c* in Arabidopsis significantly enhanced the scavenging ability of ROS and improved the tolerance of Arabidopsis to Cu. Cu induces the expression of different types of *MT* genes in plants in a concentration-dependent manner [121]. For example, Cu induces the expression of *MT1a* and *MT2b* in Arabidopsis roots and leaf phloem, and an increase in the concentration of Cu concentration leads to the upregulation of *MT2a* and *MT3* expression in mesophyll cells.

#### 5.4.4. Melatonin

Melatonin, an indole tryptamine, is widely present in animals and plants. In plants, melatonin is not only involved in growth metabolism, seed germination, root architecture, and flowering regulation but also serves as an endogenous free radical scavenger, which can enhance stress resistance [122]. Melatonin induces the expression of specific genes and increases the content of GSH and PCs to sequester excess Cu^2+^, which is transported and stored in the apoplast [123]. Zhao et al. [124] found that exogenous melatonin effectively increased the content of Pro and antioxidants in the leaves of kiwifruit (*Actinidia deliciosa*) seedlings under Cu stress, enhanced the osmotic regulation and antioxidant capacity of cells, and alleviated Cu toxicity.

## 6. Conclusions and Future Perspective

**Figure 2:**
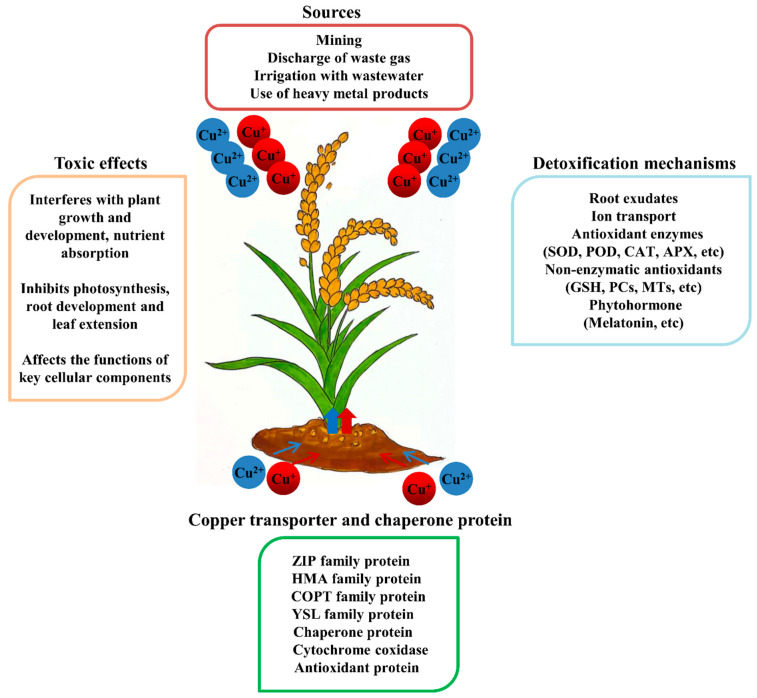
Responses and defense mechanisms of plants to copper stress.

The frequent overuse of agrochemicals is one of the primary reasons for the excessive accumulation of Cu in agricultural soil. Cu stress affects the biological diversity of soil and enzyme activity, resulting in a decrease in soil fertility and a reduction in the yield and quality of crops. This paper reviews the biological functions of Cu in plants, Cu toxicity, regulation of Cu uptake and transport, and mechanisms of Cu tolerance (Figure 2).

Since Cu plays a key role in different biochemical reactions and tissues, the regulation of Cu uptake, transport, and distribution is essential for normal plant growth. While various functional proteins are involved, the mechanisms for the interaction between different members in the gene family, physiological and biochemical action, and molecular regulatory networks remain unclear. In addition, proteins, such as HMA and NRAMP, not only regulate the homeostasis of Cu but also participate in the absorption and transport of zinc, iron, cadmium, and manganese. What is the synergistic or antagonistic relationship between Cu and other metal elements in plants? Can we discover new specific functional genes to regulate Cu homeostasis by screening mutant libraries under Cu stress? Can new types of germplasm with a high tolerance to Cu be obtained using inducible promoters to drive the expression of Cu uptake transporter genes in specific environments and organs? Can new varieties with broad-spectrum resistance to heavy metals be obtained by mining the upstream regulatory factors of HMA and NRAMP proteins and finding natural variations in plant germplasm resources that could then be used for gene editing? In-depth answers to these scientific questions will aid future soil management and enable the cultivation of plant varieties that are highly resistant to Cu.

## Figures and Tables

**Table 1:** Copper uptake and transport genes in different species.

Species	Gene	Pattern of Expression	Subcellular Localization	References
Arabidopsis (*Arabidopsis thaliana*)	*AtCOPT1*	Most tissues, roots, and reproductive tissues	Plasma membranes	[37,38]
	*AtCOPT2*	Most tissues and roots	Plasma membranes	[38,39]
	*AtCOPT3*	Reproductive tissues	Plasma membranes	[37]
	*AtCOPT5*	Most tissues, roots, and reproductive tissues	Vacuoles	[39]
	*AtCOPT6*	Reproductive tissues, xylem, and phloem vascular tissues	Plasma membranes	[39]
	*AtHMA1*	Green tissues	Chloroplast envelopes	[40]
	*AtHMA5*	Roots and flowers	Plasma membranes	[41]
	*AtHMA6*	Roots and shoots	Chloroplasts	[42]
	*AtHMA7*	Roots and flowers	Endoplasmic reticulum	[43]
	*AtHMA8*	Aboveground	Thylakoid membranes	[44]
	*AtYSL1*	Most tissues and roots	Plasma membranes	[45]
	*AtYSL2*	Most tissues, roots, and stems	Plasma membranes, vessels	[46]
	*AtYSL3*	Young leaves, roots, and stems	Plasma membranes	[46]
	*AtZIP2*	Roots	Cell membranes	[47]
	*AtZIP4*	Roots	——	[47]
Rice (*Oryza sativa*)	*OsCOPT1*	Most tissues, roots, and stems	Plasma membranes	[48]
	*OsCOPT2*	Most tissues and roots	Plasma membranes	[49]
	*OsHMA5*	Xylem of vascular bundles at the nodes, pedicels, and petioles	Plasma membranes	[41]
	*OsHMA9*	Xylem and phloem vascular tissue	Plasma membranes	[50]
	*OsYSL16*	Phloem and vascular tissue of the roots, stems, and leaves	Plasma membranes	[51]
Medicago (*Medicago truncatula*)	*MtCOPT1*	Roots	Plasma membranes	[38]
	*MtCOPT3*	Nodules	——	[38]
	*MtCOPT4*	Roots	——	[38]
	*MtCOPT5*	Roots	——	[38]
	*MtCOPT8*	Root, xylem, and phloem vascular tissues	——	[38]
Grape (*Vitis vinifera*)	*VvCTr1*	Xylem and phloem vascular tissue, leaves, and roots	Vacuole membranes	[52]
	*VvCTr2*	——	——	[53]
	*VvCTr8*	——	——	[53]
Wheat (*Triticum aestivum*)	*TaCT1*	Xylem and phloem vascular tissue, roots, and grains	Golgi apparatus	[54]
Rape (*Brassica napus*)	*BnHMA1*	Leaves	——	[55]
	*BnCOPT2*	Roots	——	[55]
Soybean (*Glycine max*)	*GmHMA8*	Leaves	Thylakoid membranes	[56]
Barley (*Hordeum vulgare*)	*HvHMA1*	Leaves and seeds	Chloroplast envelopes	[57]
	*HvYSL2*	Stems, young leaves, and root endodermis	——	[58]
Peanut (*Arachis hypogaea*)	*AhYSL3.1*	Roots, stems, young leaves, and old leaves	Plasma membranes	[59]
	*AhYSL3.2*	Roots, stems, young leaves, and old leaves	——	[59]

## Data Availability

Not applicable.
